# Patient subgroups and shifting incidence trends: an epidemiological profile of registered severe mental disorders in Quzhou, China (1969–2022)

**DOI:** 10.3389/fpubh.2026.1783400

**Published:** 2026-06-15

**Authors:** Lushan Wang, Yuefen Wan, Feipeng Chen, Chunfu Fang, Ruifang Zhao, Xianchen Jiang

**Affiliations:** 1 Quzhou Center for Disease Control and Prevention, Quzhou, Zhejiang, China; 2 Third Hospital of Quzhou, Quzhou, Zhejiang, China; 3 Quzhou Qujiang Center for Disease Control and Prevention, Quzhou, Zhejiang, China

**Keywords:** cross-sectional study, Joinpoint regression, schizophrenia, severe mental disorders, trend

## Abstract

**Background:**

Severe mental disorders (SMD) pose a significant public health challenge. Comprehensive epidemiological data are crucial for informing effective management strategies, yet detailed profiles from specific regions like Quzhou, China, remain limited.

**Methods:**

This retrospective study analyzed data from 11,589 patients with registered SMD in Quzhou, sourced from the National Information System for Psychosis. We extracted demographic, clinical, and management information. Statistical analyses included descriptive statistics, chi-square/Fisher’s exact tests to compare characteristics across six SMD categories, hierarchical cluster analysis to explore disorder groupings, and Joinpoint regression to analyze incidence trends from 1969 to 2022.

**Results:**

The cohort was 52.4% female, and predominantly had low educational attainment (84.7%with junior high school or below). Schizophrenia was the most prevalent diagnosis (66.0%). Significant differences (all *p* < 0.001) were observed in all sociodemographic and clinical variables across disorder types. Cluster analysis identified three distinct groups: (1) schizophrenia, bipolar affective disorder, and paranoid mental disorder; (2) intellectual disability with associated psychiatric disorder (a highly distinct profile); and (3) psychiatric disorders due to epilepsy and schizoaffective disorder. Joinpoint regression indicated a significant overall increase in crude incidence from 1969 to 2022, with an Average Annual Percent Change of 6.06% (95% CI: 5.04 to 7.14%), characterized by a strong rise until 2013 with an Annual Percent Change (APC) of 8.40% (95% CI: 7.58 to 9.58%), after which the trend stabilized, showing a non-significant downward trend (APC: −4.67%; 95% CI: −26.80 to 3.24%).

**Conclusion:**

This study provides a detailed epidemiological profile of SMD in Quzhou, revealing distinct patient subgroups and a recent plateau in the incidence trend. The findings underscore the need for differentiated, cluster-informed public health planning and resource allocation to address the varied needs of this population.

## 1 Introduction

Severe mental disorders (SMD) are characterized by serious symptoms that impair the patients’ social adaption and other functions and cause their failure to fully understand the reality, realize their own health condition or deal with their personal affairs (1). In China, SMD are officially concluded six major diagnostic types: schizophrenia, paranoid mental disorder, schizoaffective disorder, bipolar affective disorder, psychiatric disorders due to epilepsy, and intellectual disability with associated psychiatric disorder (2). Individuals with SMD face a mortality risk 2–3 times higher than the general population, which translates to a reduction in life expectancy of 10–20 years (3, 4). This excess morbidity and mortality are further compounded by associated social deprivation (5). Most patients require long-term treatment and care, which substantially affects their quality of life and imposes considerable emotional, social, and economic burdens on both patients and their families (6, 7). Consequently, the overall disease burden of SMD were amplified by its high incidence, frequent relapse, significant disability rates, and the profound health inequities experienced by this population (8, 9).

To improve health services for SMD, the Chinese government has implemented a series of policy measures (10). In 2005, the government launched a pilot program known as the management and treatment project for SMD (also called the 686 Project), providing financial subsidies and professional medical guidance to patients (11). The target of 686 project was patients with SMD who had a clear diagnosis, and poor patients who lived at home and whose annual household income was lower than the local average annual income level. Psychiatrists provided free and essential medicines regularly according to the disease. Doctors in township health centers or community health service centers followed up the patients regularly, checked their mental status and physical diseases, and conducted risk assessments (12). This project has proven effective in improving treatment rates, quality of life, and social function among patients, while reducing risk-related behaviors (13, 14). Due to its positive outcomes, the project was integrated into the National Basic Public Health Service Program in 2009 and expanded across the country (15). Then on 1 May 2013, China’s “Mental Health Law” went into effect, the first-ever national law on mental health and a landmark policy for protecting patient rights and improving mental health services (16).

Quzhou, located in western Zhejiang Province, features a predominantly mountainous and hilly topography. Its demographic structure, geographic conditions, and healthcare accessibility have certain regional characteristics. In 2022, the per capita GDP of Quzhou was 87,544 yuan, which closely approached the national per capita GDP of 85,698 yuan and was comparable to the average level of prefecture-level cities in China (17, 18). In this policy context, the long-term registry data from Quzhou provide a valuable window for understanding case detection, registration management, and disease spectrum changes in non-core areas. Thus, this study offers regional evidence for the international community regarding the epidemiological features and governance challenges of SMD in the context of expanding mental health services in moderately resourced settings.

## 2 Methods

### 2.1 Data sources

Our study included patients with registered Severe Mental Disorders (SMD) from Quzhou. The patients’ data were primarily managed by the Third Hospital of Quzhou, which serves as the city’s sole designated tertiary specialized psychiatric hospital. The data were extracted from the Zhejiang Provincial Severe Mental Disorder Management System. Eligible patients were diagnosed with one of the following disorders: schizophrenia (ICD-10: F20), paranoid mental disorder (F22), schizoaffective disorder (F25), bipolar affective disorder (F31), psychiatric disorders due to epilepsy (F06.8), or intellectual disability with associated psychiatric disorder (F7X.1/F7X.8). All patients were diagnosed by qualified medical institutions, registered in the system before December 31, 2022. Registered SMD patients subsequently received regular follow-up management through local community services. Exclusion criteria comprised patients who were deceased, lost to follow-up, not under active management, or receiving only temporary assistance. Population data were obtained from the statistical bulletin of Quzhou Municipal Bureau of Statistics.

### 2.2 Statistical analysis

Statistical analysis was performed by R software (Version 4.4.1) and Joinpoint Regression software (Version 5.4.0). A two-sided *p* value <0.05 was considered statistically significant.

Descriptive statistics were used to summarize patient characteristics. Categorical variables are presented as frequency and Proportion. Differences in the distribution of characteristics across the six SMD categories were assessed using the Chi-square test, however, Fisher’s exact test was employed when the expected cell count in any contingency table was less than 5. Cluster analysis was conducted to group the six SMD types based on the following patient characteristics: gender, age group, education level, marital status, disease duration, and family history of mental disorders. These variables were selected because they are routinely collected in the registry and are known to differ across SMD diagnoses. Raw variables were first converted to Z-scores. A dissimilarity matrix was then computed using squared Euclidean distance. Ward’s minimum variance method was applied to this matrix to generate the primary clustering solution and corresponding dendrogram. The robustness of the cluster structure was evaluated in a sensitivity analysis using the average linkage method. The optimal number of clusters was determined through visual inspection of the dendrogram and clinical interpretability.

Joinpoint regression was used to analyze trends in the incidence of SMD (annual new cases per 10 million population) from 1969 to 2022. This model identifies significant inflection points where trends change direction. The annual percent change (APC) for each segment and the average annual percent change (AAPC) for the entire period was calculated, each with a corresponding 95% confidence interval (CI). The final model was selected using a permutation test. All APCs and AAPCs are reported with 95% confidence intervals. For trends with confidence intervals crossing zero, we interpret them as non-significant changes.

## 3 Results

### 3.1 Demographic distribution

A total of 11,589 patients with SMD were registered in Quzhou in the end of 2022. Their baseline demographic and clinical characteristics are summarized in Table 1. The cohort comprised 52.4% (6,073/11,589) females. The majority (63.9%) had an age of onset between 15 and 44 years. Overall educational attainment was low, with 84.7% having completed junior high school or less. More than half (54.4%) were married, and a substantial proportion (76.0%) had a disease duration exceeding 10 years. In contrast, only 6.1% reported a positive family history of mental disorders.

**Table 1:** Demographic characteristics of the registered SMD patients (*N* = 11,589).

Variable	Frequency	Proportion (%)
Gender		
Male	5,516	47.6
Female	6,073	52.4
Age		
<15	81	0.7
15–29	3,746	32.3
30–44	3,622	31.6
45–59	2,881	24.9
≥60	1,219	10.5
Mean (S.D.)	52.5 (14.9)	
Median (IQR)	53 (42–63)	
Education level		
Illiterate	3,030	26.2
Primary school	3,341	28.8
Junior school	3,447	29.7
High school or secondary school	1,090	9.4
College degree or above	337	2.9
Unknown	344	3.0
Marital status		
Unmarried	3,928	33.9
Married	6,301	54.4
Widowed	575	5.0
Divorced	737	6.4
Unknown	48	0.4
Disease duration		
<3	429	3.7
3–	493	4.3
5–	1861	16.1
10–	3,712	32.0
≥20	5,094	44.0
Mean (S.D.)	20.1 (12.6)	
Median (IQR)	17.8 (10.3–27.7)	
Family history of mental disorders		
Yes	710	6.1
No	10,815	93.3
Unknown	64	0.6

### 3.2 Distribution across the six major SMD categories

The distribution of gender, age, education, marital status, disease duration, and family history varied considerably across the six SMD categories (Table 2). Schizophrenia was the most prevalent diagnosis, comprising 66.0% (*n* = 7,646) of cases, with the following distribution for the remaining disorders: bipolar affective disorder (13.6%), intellectual disability with associated psychiatric disorder (12.6%), psychiatric disorders due to epilepsy (5.1%), schizoaffective disorder (1.7%), and paranoid mental disorder (1.0%).

**Table 2:** Demographic characteristics of the registered SMD patients by six categories (*N* = 11,589, *N* (%)).

Variable	Schizophrenia (*n* = 7,646)	Intellectual disability with associated psychiatric disorder (*n* = 1,466)	Bipolar affective disorder (*n* = 1,573)	Psychiatric disorders due to epilepsy (*n* = 595)	Paranoid mental disorder (*n* = 114)	Schizoaffective disorder (*n* = 195)	Chi-square	*P* value
Gender							143.198	<0.001
Male	3,485 (45.6)	866 (59.1)	672 (42.7)	357 (60.0)	51 (44.7)	85 (43.6)		
Female	4,161 (54.4)	600 (40.9)	901 (57.3)	238 (40.0)	63 (55.3)	110 (54.6)		
Age							534.116	<0.001
<15	13 (0.2)	53 (3.6)	2 (0.1)	12 (2.0)	0 (0.0)	1 (0.5)		
15–29	2,534 (33.1)	545 (37.2)	393 (25.0)	193 (32.4)	25 (21.9)	56 (28.7)		
30–44	2,614 (34.2)	385 (26.3)	371 (23.6)	200 (33.6)	24 (21.1)	68 (34.9)		
45–59	1845 (24.1)	355 (24.2)	444 (28.2)	151 (25.4)	31 (27.2)	55 (28.2)		
≥60	640 (8.4)	128 (8.7)	363 (23.1)	39 (6.6)	34 (29.8)	15 (7.7)		
Mean (S.D.)	53.1 (13.9)	45.8 (16.4)	55.9 (16.5)	48.1 (14.4)	60.0 (14.7)	57.5 (13.8)		
Median (IQR)	53 (44–63)	47 (33–57)	57 (45–69)	50 (39–57)	59 (50–71)	58 (48–68)		
Education level							1065.993	<0.001
Illiterate	1,618 (21.2)	846 (57.7)	331 (21.0)	174 (29.2)	26 (22.8)	34 (17.4)		
Primary school	2,213 (28.9)	364 (24.8)	448 (28.5)	215 (36.1)	31 (27.2)	70 (35.1)		
Junior school	2,509 (32.8)	188 (12.8)	480 (30.5)	157 (26.4)	40 (35.1)	73 (37.4)		
High school or secondary school	842 (11.0)	18 (1.2)	185 (11.8)	27 (4.5)	8 (7.0)	10 (5.1)		
College degree or above	259 (3.4)	3 (0.2)	60 (3.8)	6 (1.0)	5 (4.4)	4 (2.1)		
Unknown	205 (2.7)	46 (3.1)	69 (4.4)	16 (2.7)	4 (3.5)	4 (2.1)		
Marital status							846.247	<0.001
Unmarried	2,373 (31.0)	939 (64.1)	307 (19.5)	240 (40.3)	31 (27.2)	38 (19.5)		
Married	4,290 (56.1)	425 (29.0)	1,078 (68.5)	302 (50.8)	64 (56.1)	142 (72.)		
Widowed	389 (5.1)	58 (4.0)	91 (5.8)	18 (3.0)	11 (9.7)	8 (4.1)		
Divorced	567 (7.4)	30 (2.1)	91 (5.8)	35 (5.9)	8 (7.0)	6 (3.1)		
Unknown	27 (0.4)	14 (1.0)	6 (0.4)	0 (0.0)	0 (0.0)	1 (0.5)		
Disease duration							247.200	<0.001
<3	214 (2.8)	106 (7.2)	82 (5.2)	14 (2.4)	11 (9.7)	2 (1.0)		
3–	278 (3.6)	95 (6.5)	89 (5.7)	20 (3.4)	6 (5.3)	5 (2.6)		
5–	1,154 (15.1)	231 (15.8)	343 (21.8)	97 (16.3)	16 (14.0)	20 (10.3)		
10–	2,635 (34.5)	337 (23.0)	473 (30.1)	170 (28.6)	41 (36.0)	56 (28.7)		
≥20	3,365 (44.0)	697 (47.5)	586 (37.3)	294 (49.4)	40 (35.1)	112 (57.4)		
Mean (S.D.)	19.8 (11.5)	22.1 (15.9)	18.5 (13.3)	21.6 (13.0)	18.1 (12.9)	23.1 (11.6)		
median (IQR)	18.0 (10.9–26.7)	18.6 (8.9–34.3)	14.7 (8.3–26.3)	19.7 (10.8–31.0)	14.3 (7.7–26.7)	22.5 (15.7–29.7)		
Family history of mental disorders							55.068	<0.001
Yes	435 (5.7)	148 (10.1)	98 (6.2)	17 (2.9)	4 (3.5)	8 (4.1)		
No	7,165 (93.7)	1,308 (89.2)	1,470 (93.4)	576 (96.8)	110 (96.5)	186 (95.4)		
Unknown	46 (0.6)	10 (0.7)	5 (0.3)	2 (0.3)	0 (0.0)	1 (0.5)		

**Figure 1:**
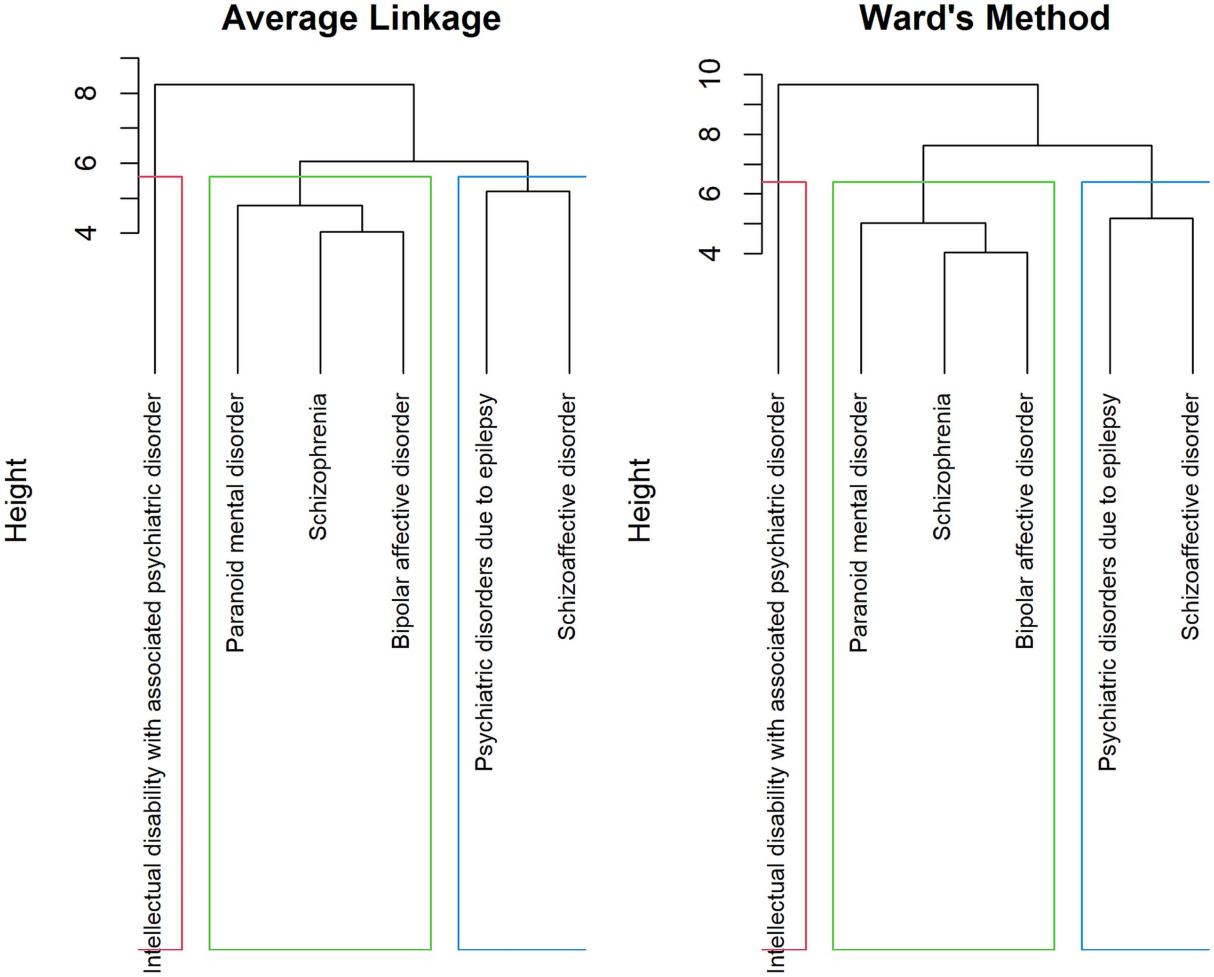
Profiles of three distinct clusters of SMD patients based on cluster analysis.

A cluster analysis of the SMD categories based on demographic profiles yielded three distinct groups (Figure 1). Ward’s method identified the following clusters: Cluster 1 comprised schizophrenia, bipolar affective disorder, and paranoid mental disorder; Cluster 2 consisted solely of intellectual disability with associated psychiatric disorder; and Cluster 3 included psychiatric disorders due to epilepsy and schizoaffective disorder. The same three-cluster structure was reproduced using average linkage, supporting its robustness.

### 3.3 Joinpoint analysis from 1969 to 2022

**Figure 2:**
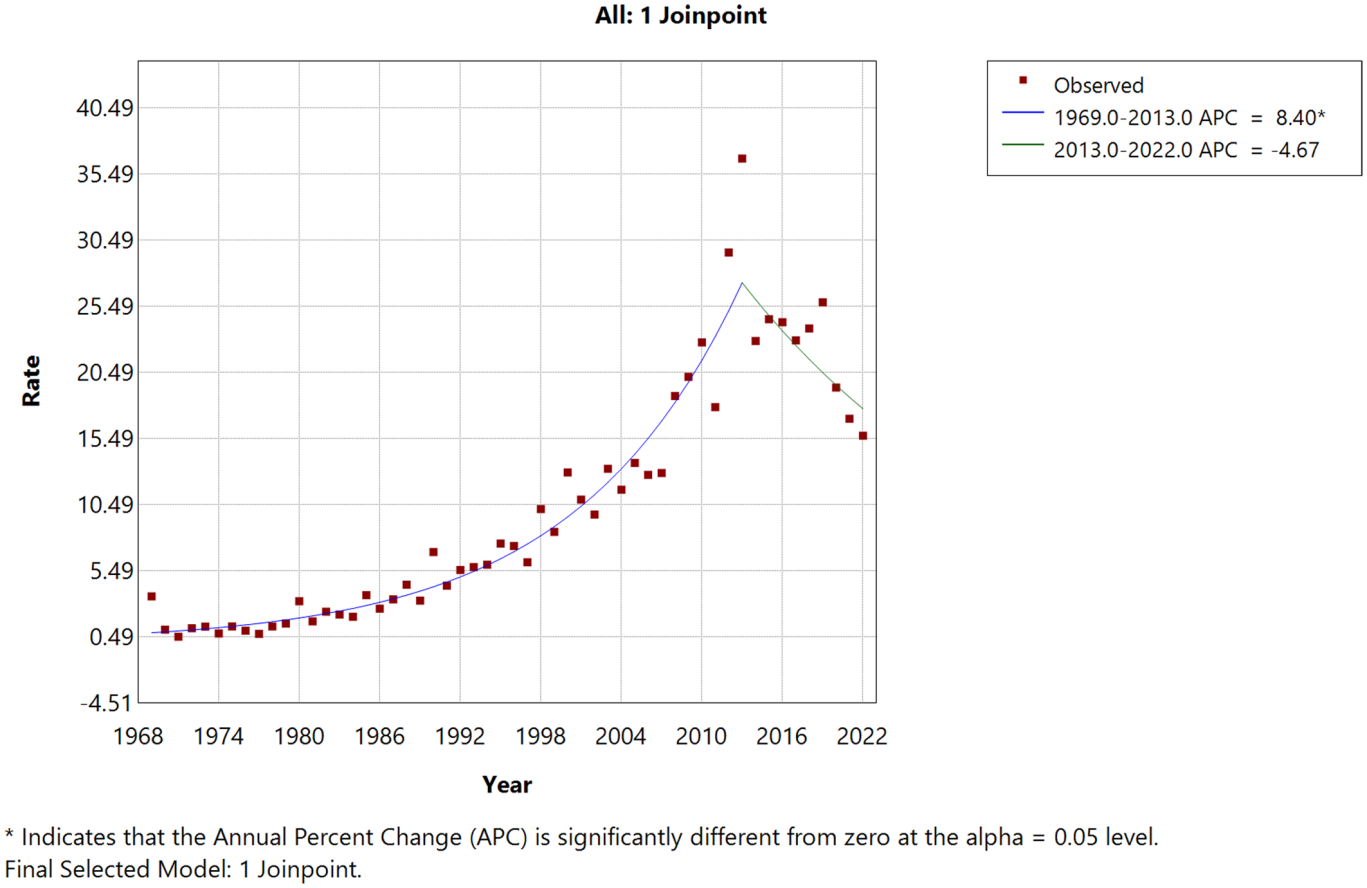
Trend of SMD by Joinpoint Regression (1 Joinpoint), 1969–2022.

The crude incidence of SMD showed an overall increasing trend from 1969 to 2013, after which it appeared to stabilize (Figure 2). Joinpoint regression confirmed an overall upward trend from 1969 to 2022, with an AAPC of 6.06% (95% CI: 5.04 to 7.14). A period of stronger increase was observed from 1969 to 2013, with an APC of 8.40% (95% CI: 7.58 to 9.58). From 2013 to 2022, the trend showed a non-significant change (APC: −4.67%; 95% CI: −26.80 to 3.24).

## 4 Discussion

This study revealed that registered SMD patients were typically young adults at onset, had low education levels, and long illness durations. Schizophrenia was the most common diagnosis among six types. Cluster analysis identified three distinct patient subgroups. Furthermore, the long-term trend showed an initial historical increase, followed by a recent plateau. This study has several unique features. The 50 + year timeframe is longer than most previous SMD reports from China. The cluster analysis provides a data-driven grouping of the six official SMD categories, which has not been done before in this region. Moreover, Quzhou is representative of many inland Chinese cities with moderate economic development. Therefore, our findings may be informative for other regions facing similar resource constraint.

Our findings confirm that schizophrenia is the most prevalent SMD diagnosis, which aligns with previous reports from other regions of China (19–21). However, the low reported rate of positive family history (6.1%) contrast to the results of previous studies (22–24). This difference is probably caused by under-reporting from stigma, or failure to spot cases in families (25).

Beyond this general pattern, our cluster analysis provides a novel, data-driven grouping of the six SMD categories. Cluster 1 includes schizophrenia, bipolar affective disorder and paranoid mental disorder. These three conditions share similar sociodemographic profiles. Despite their clinical differences, these disorders share episodic severe symptoms, high relapse rates and major functional impairment (20, 26, 27). Therefore, they require integrated public health strategies that ensure regular medication, prevents relapse, and supports mental and social recovery within the community (28). Cluster 2 only covers intellectual disability with related psychiatric disorders. This disorder is characterized by prominent early onset neurocognitive deficit (29). The group demands a lifelong support system focused on special education, custodial care and family support (30). Cluster 3 combines psychiatric disorders due to epilepsy with schizoaffective disorder. These two conditions share similar clinical complexity. Their management requires integrated collaboration between neurology and psychiatry for comprehensive assessment and intervention (31, 32). While personalized care strategies derived from this clustering remain preliminary and require further validation, the findings hold potential to inform mental health services and may help refine community case management and lifecycle support services.

The Joinpoint regression results revealed a notable shift in the long-term incidence trend. From 1969 to 2013, the crude incidence of SMD increased significantly. The early upward trend is consistent with findings from other studies (13, 14). This period was characterized by rapid urbanization and modernization, which heightened life pressures and job competition, contributing to the observed increase in SMD incidence (10, 33). After 2013, however, the trend appeared to stabilize, with an APC of −4.67% and a 95% CI ranging from −26.80 to 3.24%. The wide confidence interval means that we cannot claim a statistically significant decline. The observed pattern may represent a true stabilization or plateau, but it could also be influenced by other factors, such as changes in registration practices. Several possible explanations for the post-2013 stabilization deserve consideration, although they remain speculative. First, the implementation of China’s Mental Health Law in May 2013 may have standardized diagnostic procedures and case registration, potentially leading to more accurate but not necessarily increased reporting. Second, the 686 Project had achieved nationwide coverage by 2009, and by 2013, the backlog of previously undiagnosed or untreated cases might have been largely absorbed, bringing the annual incidence closer to the true underlying rate. Third, there is evidence from randomized controlled trials that psychological interventions can have preventive effects in both young people and adults, and that these can be cost-effective (34, 35). Such preventive efforts may have contributed, at least in part, to the stabilization of incidence in recent years. Further studies with longer follow-up and more detailed data on service utilization are needed to clarify the exact drivers of this post-2013 trend.

## 5 Conclusion

The study found that registered SMD patients were typically young, low education, and long illness durations. Schizophrenia was the most common diagnosis. Cluster analysis identified three distinct patient subgroups. The incidence trend showed a significant increase from 1969 to 2013 and then showed signs of stabilization in recent years. These findings have direct implications for regional public health strategy. They provide a cluster-based framework to support rational resource allocation and inform the development of healthcare plans.

## Data Availability

The data analyzed in this study is subject to the following licenses/restrictions: the datasets are not publicly available due to patient privacy, but de-identified data and analysis code are available from the corresponding author on reasonable request. Requests to access these datasets should be directed to XJ, jxc820119@163.com.
